# pH effect on strain-specific transcriptomes of the take-all fungus

**DOI:** 10.1371/journal.pone.0236429

**Published:** 2020-07-30

**Authors:** Kévin Gazengel, Lionel Lebreton, Nicolas Lapalu, Joëlle Amselem, Anne-Yvonne Guillerm-Erckelboudt, Denis Tagu, Stéphanie Daval

**Affiliations:** 1 IGEPP, INRAE, Institut Agro, Univ Rennes, Le Rheu, France; 2 AgroParisTech, INRAE, Université Paris-Saclay, BIOGER, Thiverval-Grignon, France; 3 INRAE, Université Paris-Saclay, URGI, Versailles, France; University of Nebraska-Lincoln, UNITED STATES

## Abstract

The soilborne fungus *Gaeumannomyces tritici* (*G*. *tritici*) causes the take-all disease on wheat roots. Ambient pH has been shown to be critical in different steps of *G*. *tritici* life cycle such as survival in bulk soil, saprophytic growth, and pathogenicity on plants. There are however intra-specific variations and we previously found two types of *G*. *tritici* strains that grow preferentially either at acidic pH or at neutral/alkaline pH; gene expression involved in pH-signal transduction pathway and pathogenesis was differentially regulated in two strains representative of these types. To go deeper in the description of the genetic pathways and the understanding of this adaptative mechanism, transcriptome sequencing was achieved on two strains (PG6 and PG38) which displayed opposite growth profiles in two pH conditions (acidic and neutral). PG6, growing better at acidic pH, overexpressed in this condition genes related to cell proliferation. In contrast, PG38, which grew better at neutral pH, overexpressed in this condition genes involved in fatty acids and amino acid metabolisms, and genes potentially related to pathogenesis. This strain also expressed stress resistance mechanisms at both pH, to assert a convenient growth under various ambient pH conditions. These differences in metabolic pathway expression between strains at different pH might buffer the effect of field or soil variation in wheat fields, and explain the success of the pathogen.

## Introduction

The filamentous fungus *Gaeumannomyces tritici* (*G*. *tritici*) is an ascomycete of large economic importance due to its devastating impact on cereal plants in temperate climates. The take-all disease caused by this fungus affects the roots of the host plants by blocking the conductive tissues and reducing water uptake. Serious infections under favorable conditions can result in decreased yields of up to 40%-60% [1].

*G*. *tritici* populations are divided into two major genetically different groups (G1 and G2) which are known to coexist at the field scale in pluri-annual wheat monoculture experiments [2]. Ratios of G1 to G2 are different due to wheat crop history and disease level. G1 strains are more frequent in the first year of wheat monoculture, whereas G2 strains increase and reach a peak after three to five years corresponding to the maximum of take-all symptoms [3]. Furthermore, *in vitro* plant assays showed that G2 strains are slightly more aggressive than the G1 [4].

As most of soilborne pathogenic fungi, *G*. *tritici* develops strategies to adapt to the ambient environmental factors all along its life cycle: survival in bulk soil, hyphal growth in soil during the saprophytic phase, and infection of host plants during pathogenic phase. This is particularly true concerning the pH factor. Soil pH is a factor influencing the take-all severity, whether modified by nitrogen supply [5] or by microbial communities interacting with *G*. *tritici* and wheat [6], leading to a more acidic rhizosphere unfavorable to *G*. *tritici* [7]. As a consequence, the pH- signaling pathway (Pal), characteristic of the fungal kingdom, has been shown to operate in *G*. *tritici* [8]. This Pal pathway, first identified in *Aspergillus nidulans* [9], is composed of six proteins (palA, palB, palC, palF, palH, palI) which conduct pH signal to the transcription factor pacC [10]. Three forms of pacC exist: the inactive full-length pacC form predominates in acidic conditions whereas, in neutral-to-alkaline conditions, two proteolytic cleavages (the first one pH-dependent) enable pacC to be functional as a repressor of acid-expressed genes and an activator of neutral-to-alkaline-expressed genes [10].

Within *G*. *tritici* species, evidence of intraspecific variability in pH sensitivity was demonstrated: some strains grew better at neutral pH and other at acidic pH [11]. More precisely, G1 and G2 strains are known to respond differently to the pH factor: whereas G1 and G2 strains have similar growth rate profile on acidic medium, G2 strains present a significantly better growth rate on neutral medium [11]. The mechanisms underlying the differential response of *G*. *tritici* strains to the pH variations of the environment are not yet elucidated but extracellular pH has been shown to regulate *G*. *tritici* gene expression involved in pathogenesis and saprophytic growth [8], in an original strain-specific way. Thus, the transcription factor pacC has been suspected to potentially play a role in pathogenesis through the regulation of expression of some pathogenesis-related genes that contained pacC binding sites (5‘-GCCARG-3’). This is true for *Penicillium expansum and Penicillium digitatum* in which pacC mutants are affected in their pathogenicity towards pear/apple and citrus fruits, respectively [12,13]. The ability of strains within the *G*. *tritici* species to fine tune gene expression in response to the soil pH could affect growth rate leading to diverse (i) capacity of saprophytic growth (survival and development in bulk soil), (ii) capacity of surface roots’ colonization (first part of the pathogenic phase), and (iii) capacity of penetrating the roots (infection phase).

The aim of this study is to decipher the effect of ambient pH on *G*. *tritici* gene expression patterns and to test the hypothesis of the link between pH perception and growth ability in the saprophytic growth and colonization of roots surface. Herein, two *G*. *tritici* strains (PG6 and PG38), differing in their growth profile in function of the ambient pH and their group (G1 for PG6 and G2 for PG38), were selected and used for an RNA-Seq analysis under acidic or neutral pH conditions: pH 4.6 to mimic the value commonly found in soils and known to be unfavorable to *G*. *tritici* [14], and pH 7.0 known as the optimal value for *G*. *tritici* [5,15]. By this transcriptomics analysis, we showed that major metabolic and physiological changes associated with ambient pH and with pH-dependent growth of two strains occurred, and a focus was performed on the differentially expressed genes (DEGs) potentially involved in pH-dependent growth ability.

## Materials and methods

### Fungal strains and culture conditions

*G*. *tritici* strains used in this study (Table 1) were stored as potato dextrose agar (PDA) explants immersed in 10% glycerol at 4°C for long-term preservation. Prior to inoculation, each strain was cultured twice for 7 days at 20°C in the dark on autoclaved non-buffered (pH 5.6) Fåhraeus medium [16], the composition of which is described in the S1 Table [8]. Two Fåhraeus media buffered at pH 4.6 (A for acidic pH) or 7.0 (N for neutral pH), with different ratios of citrate / phosphate solutions (S1 Table), were then used for fungal mycelium growth measurement. pH was checked in all the media after autoclaving (115°C, 20 min). An Isopore 0.22 μm pore-size sterile membrane (Millipore, Molsheim, France) was laid on the agar surface to force mycelium to grow on top of the medium and to facilitate its sampling for further RNA extractions. Five mm diameter plugs of mycelium were removed from the edge of a colony grown twice the non-buffered medium. One plug per plate was laid on buffered Fåhraeus media (A or N), in the centre of Petri dishes, and the plates, covered by the polycarbonate filter, were incubated for 7 days at 20°C in the dark. Two orthogonal diameters of the colony were measured in each condition 7 days after inoculation. For each pH condition and strain, three plates were used, and three independent experiments were performed. Mycelium growth was compared using the Wilcoxon rank sum test.

**Table 1:** Origin of *Gaeumannomyces tritici* isolates used in this study.

Isolate	Original nomenclature	Molecular characterization (G1/G2)	Geographical origin / Isolation date	Source or reference	Host of origin
PG6	1125–6	G1	Germany / 1997	Monsanto collection	Wheat
PG38	82/02	G2	France (Pacé) / 2002	Lebreton et al., 2007 [3]	Wheat

### RNA extraction from fungal mycelium grown on buffered media

After incubation at 20°C for 7 days, all the mycelium grown on polycarbonate membrane was collected. After sampling, the pH of each plate was measured to verify that it was still buffered at the desired initial pH levels. The mycelia from 3 plates were pooled and ground to powder with a pestle in liquid nitrogen-chilled mortars with Fontainebleau sand. Total RNA was extracted in 1 mL of Trizol (Invitrogen, Paisley, UK) and contaminating DNA was removed by using the RNase-free RQ1 DNase (Promega Corp., Madison, WI, USA) according to the manufacturer’s instructions. The RNA purity and quality were assessed with a Bioanalyser 2100 (Agilent Tech. Inc., La Jolla, CA, USA) and quantified with a Nanodrop (Agilent).

### Library construction, RNA-sequencing, and quality control

For each of the twelve samples (2 strains * 2 pH conditions * 3 biological replicates), sequencing libraries were generated using 2 μg of total RNA per sample with the TruSeq RNA sample preparation protocol from Illumina according to the manufacturer’s recommendations. Tags were added to each sample for identification. The sequencing reaction was performed using the Illumina HiSeq v3 chemistry on a HiSeq 2000 in 100 bp single read run according to the manufacturer’s recommendations (GATC Biotech, Konstanz, Germany). Sequence data quality control was evaluated using the FastQC program. Adapter sequences were removed using Flexbar, and Sickle was used to clean bases with substandard quality (PHRED 28) before removing reads under thirty bases length. Raw reads are available at the European Nucleotide Archive database system under the project accession number PRJEB34060.

### Read mapping to the reference genome and transcript counting

STAR v2.5.2a_modified [17] was used to align the reads to the published genome of *G*. *tritici* strain (https://fungi.ensembl.org/Gaeumannomyces_graminis/Info/Index). This available reference sequenced genome is from the R3-111a-1 *G*. *tritici* strain, different from the strains used in our study [15,18]. FeatureCounts v1.6.0 [19] was used to count the number of reads on each annotated *G*. *tritici* gene, giving the raw counts. Genes with low count levels (under 1 count per million of mapped reads in three samples at least) were removed from the data.

### Differential expression analysis and gene clustering

DESeq2 R package [20] was used to product lists of DEGs between two experimental conditions. This program has its own normalization method ‘Relative Log Expression’ (RLE) and needs to have raw counts as input. The p-values were adjusted to control multiple testing using the Benjamini and Hochberg’s method. Genes with an adjusted p-value < 0.05 were considered as significantly differentially expressed between conditions.

The clustering of gene expression profiles was performed using HTSCluster R package [21] by making groups of co-expressed genes on the DESeq2 normalized counts.

### Gene Ontology term (GO-term) enrichment

GO enrichment analysis of DEGs was achieved with TopGO R package [22] using weight01 algorithm, Fisher's exact statistic test and a nodeSize parameter set to 5 (to remove enriched GO-term with less than five genes in the genome). GO-terms for each gene were first imported from BioMart database (Ensembl Fungi) via Blast2GO software (https://www.blast2go.com). For each GO category (Molecular Function, Cellular Component and Biological Process), Top 10 Enriched GO-terms (p-value < 0.05), enrichment ratios (> 5), and number of genes under each enriched GO-term were represented using ggplot2 R package.

### Quantitative real-time PCR (qRT-PCR) validation

The expression levels of 8 selected DEGs were determined by qRT-PCR to confirm the results of RNA-Seq analysis. Total RNA from *G*. *tritici* mycelium was reverse transcribed with a set of two external RNA quality controls as previously described [8,23]. Briefly, for all the strains and the pH conditions, 750 ng of total RNA from fungi were mixed with known quantities of the two external controls. Reverse transcription was carried out in 30 μL containing 375 ng of random primers, 1 X ImPromII reaction buffer, 3 mM MgCl_2_, 125 μM of each dNTP, 30 U of RNasin Ribonuclease Inhibitor and 1.5 μL of ImProm-II^TM^ (Promega). The following parameters were applied: 5 min at 25°C, 1 h at 42°C and 15 min at 70°C. Reactions without RNA or without reverse transcriptase were performed as negative controls. The oligonucleotides designed with the Primer 3 software are described in S2 Table. Quantitative PCR reactions (20 μL) containing 1 μl of cDNA, 0.4 μM of each primer and 1 X SybrGreen I Master (Roche) were performed on the LightCycler^®^ 480 Real-Time PCR System (Roche). The quantitative PCR profile consisted of an initial denaturation at 95°C for 5 min, followed by 45 cycles of 95°C for 15 sec and hybridization-elongation temperature for 40 sec (S2 Table). A dissociation stage was applied at the end of the PCR to assess that each amplicon generated was specific. Moreover, each specific amplicon was sequenced (Genoscreen, Lille, France) to confirm it corresponded the expected sequence. The expression levels of transcripts were normalized using external RNA controls [23,24] from three independent biological replicates, each with three technical PCR replicates. Data were analysed using the ANOVA procedure of the R statistical analysis software.

## Results and discussion

### Effect of pH on growth

**Fig 1:**
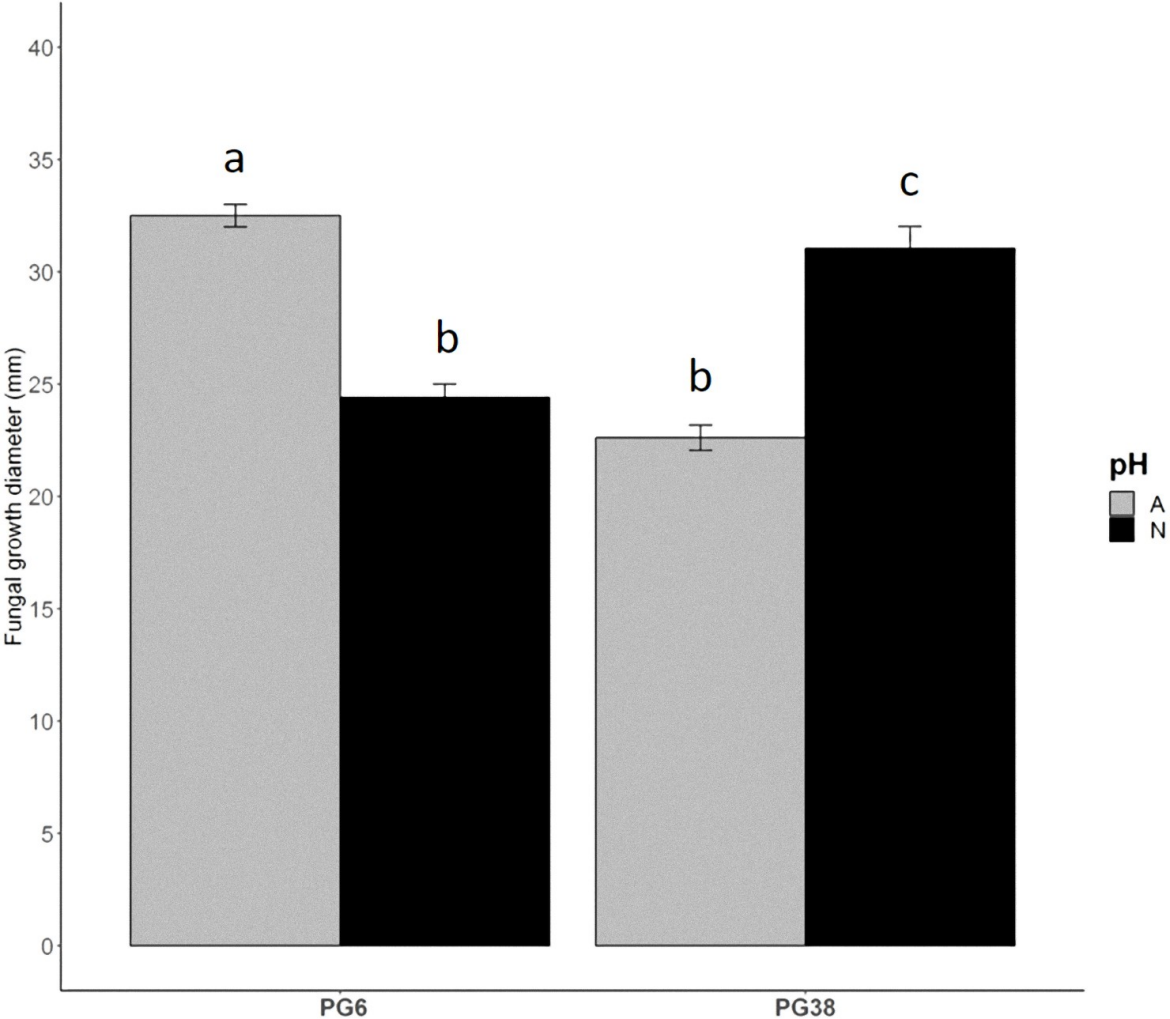
Mycelium growth of *G*. *tritici* strains as a function of medium pH. PG6 and PG38 *G*. *tritici* strains were grown at 20°C in the dark on media buffered at pH 4.6 (A) or 7.0 (N). The *G*. *tritici* plugs were laid on medium covered with a polycarbonate filter allowing the mycelium sampling. The colony diameters were measured after 7 days of incubation. Each value is the mean of three biological replicates and three technical replicates. Error bars represent standard errors of the means. Conditions with different letters are statistically different according to the Wilcoxon rank sum test (P<0.05). Strains are depicted on the x-axis and fungal growth diameter in mm is on the y-axis.

The two *G*. *tritici* strains (PG6 and PG38) were grown on media buffered at pH 4.6 (A) or 7.0 (N) in the dark. After 7 days, measurement of the pH of each plate showed that the media were still buffered at the expected initial pH. This shows that a local modulation of the ambient pH did not occur, and that this experimental set-up did allow for a pH-specific growth study. Then, two orthogonal diameters of the colony were measured in each condition (Fig 1). Both strains grew at both pH levels but displayed two different growth profiles. PG6 showed a significantly higher mycelium growth at the acidic pH compared to the neutral condition. On the contrary, PG38 grew significantly better in a neutral medium than in an acidic environment. These growth rate profiles are representative of the interspecific variability of *G*. *tritici* populations [8,11]. The growth profiles were different between strains according to the pH. This made relevant a transcriptomics study to decipher the molecular basis that could explain in part these two distinct phenotypes.

### Overview, mapping and counting of the RNA-Seq data

RNA-Seq expression profiling was performed on the two strains (PG6 and PG38) growing on two different pH (4.6 or 7.0). The three biological replicates of each condition were sequenced in one pool of 12 tagged libraries on an Illumina HiSeq 2000 in 100 bases single-read mode. From 11,956,072 to 20,507,372 raw reads were obtained per sample (Table 2). After removing adapters and low quality bases (< Q28), 91.0 to 92.6% of the raw reads were kept with a minimum of length set to 30 bases. Thus, a total of 10,957,477 to 18,783,248 cleaned reads per sample were generated from the different RNA libraries. After read trimming, 78.2 to 86.1% of the initial raw data were uniquely mapped to the *G*. *tritici* genome. Reads with multiple location (0.2 to 1.0%) and too short alignments (6.0 to 13.3%) were removed. At the end, 70.2 to 78.5% of the raw reads were kept. We removed the raw counts with less than one count per million of reads in at least three samples. We kept 11,041 genes among the 14,744 contained in the *G*. *tritici* genome. Thus, the accuracy and quality of the sequencing data were sufficient for further analysis

**Table 2:** Overview of number of raw, cleaned, mapped, and counted reads from the different samples.

Sample name	Library for each replicate	Number of total raw reads	Reads after cleaning and trimming (l30, q28)				
			Number of cleaned reads	Number of uniquely mapped reads ^1^	%	Number of counted reads ^2^	%
PG6 / A	1	15,659,005	14,506,452	12,640,293	80.7	11,430,087	73.0
	2	12,994,429	11,874,270	10,225,348	78.7	9,126,369	70.2
	3	15,355,211	14,211,595	12,249,379	79.8	10,991,518	71.6
PG6 / N	1	19,466,804	17,734,183	15,223,234	78.2	13,666,878	70.2
	2	16,798,714	15,407,637	13,261,468	78.9	12,039,586	71.7
	3	17,529,036	16,035,463	13,869,536	79.1	12,636,834	72.1
PG38 / A	1	17,641,336	16,047,490	14,867,129	84.3	13,654,777	77.4
	2	17,583,033	16,111,331	15,082,270	85.8	13,762,219	78.3
	3	14,107,598	12,956,289	12,150,819	86.1	11,071,449	78.5
PG38 / N	1	20,507,372	18,783,248	17,241,029	84.1	15,636,718	76.2
	2	17,616,201	16,206,580	15,024,729	85.3	13,707,008	77.8
	3	11,956,072	10,957,477	9,938,207	83.1	9,012,954	75.4

The percentages are given from initial raw reads.

^1^ Total number of reads mapped to uniquely locations in the *G*. *tritici* genome with STAR.

^2^ Total number of reads counted with FeatureCounts.

A: acidic pH and N: neutral pH.

### Validation and clustering of the RNA-Seq data

We looked for differential gene expression between the different samples and normalized DESeq2 counts of all samples were plotted on a PCA to estimate the variability of the experiments and the biological conditions (S1 Fig). 86% of the variance was represented on the plot. We confirmed that the three replicates of each experimental condition were largely clustered together, which validate our RNA-Seq experiment. The x-axis (57%) separated clearly the two strains whereas the y-axis (29%) was representative of the pH effect.

**Fig 2:**
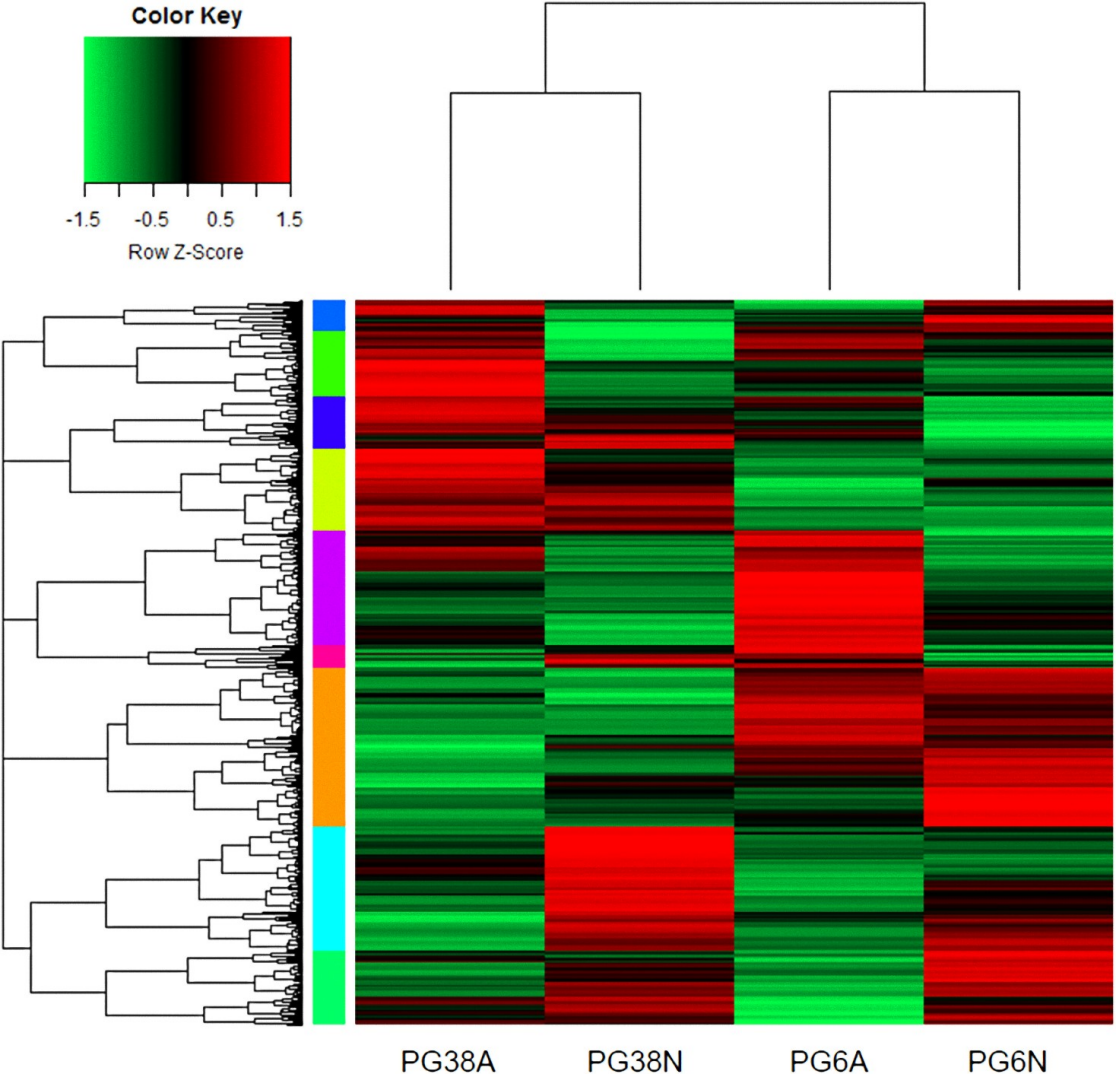
Overview of gene expression. Gene expression levels were displayed from green (downregulated) to red (upregulated). Colored bars on the left of the heatmap mark distinct major branches in the clustering tree grouping genes with similar expression pattern. Each row corresponded to a single gene and each column to the mean of the three biological repetitions of one experimental condition. The heatmap was generated with scripts based on heatmap.2 function as available in the “gplots” R package. A: acidic pH and N: neutral pH.

As biological replicates were homogeneous, gene expression level of the four experimental conditions was calculated based on the means of the three replicates of each biological condition (Fig 2). Expression profiles were clearly different between conditions, identifying groups of up-regulated (red) and down-regulated (green) genes. Gene expression was affected by the pH and/or by the strain. A clear separation was seen first between the strains, and secondly between pH inside each strain.

**Fig 3:**
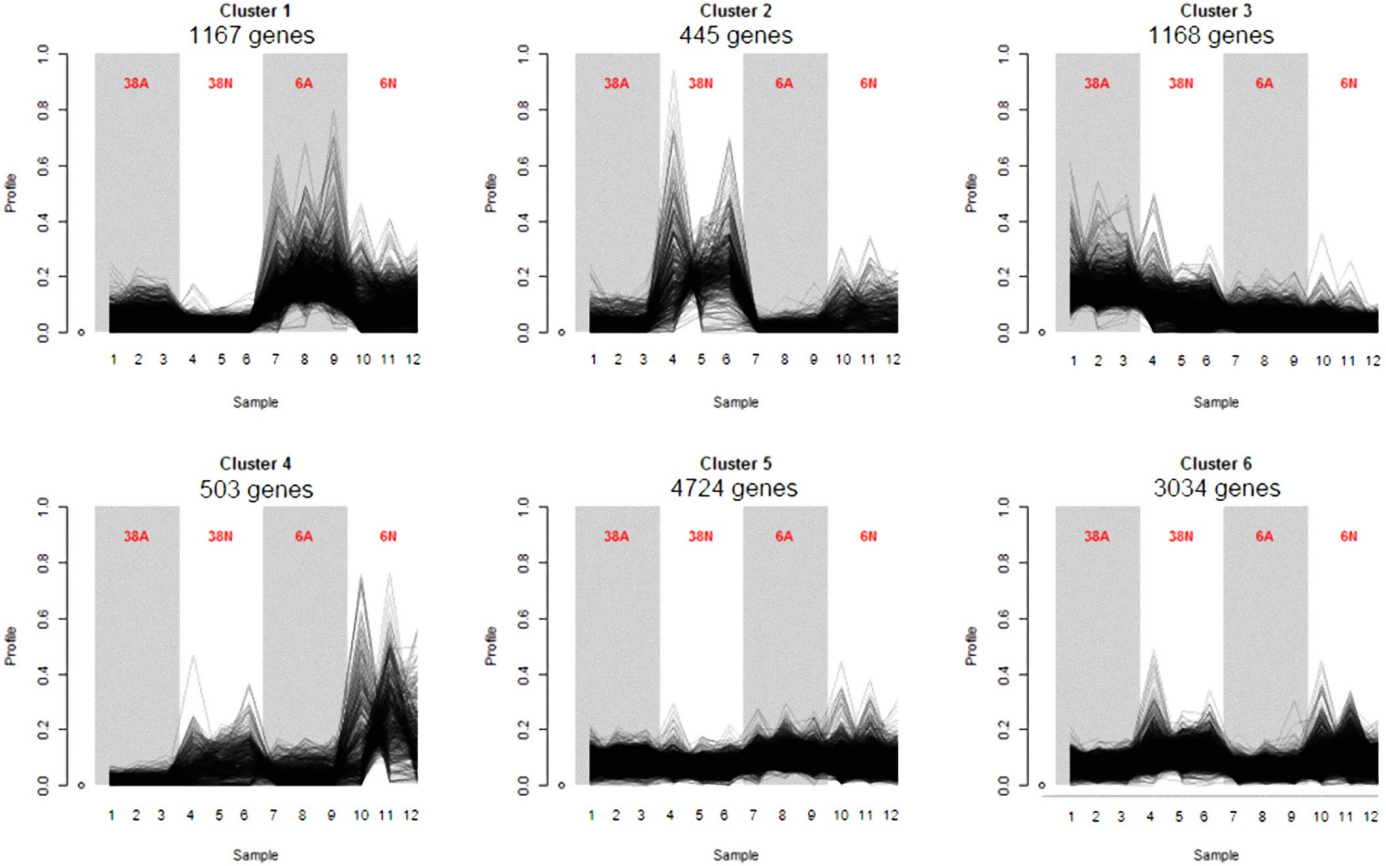
Co-expression clusters. HTSCluster was used to cluster expression data with Poisson Mixture Models. The number of genes assigned to a particular cluster is indicated below the cluster name. Grey and white backgrounds are for acidic and neutral pH, respectively. Each of the 12 samples is depicted on the x-axis and each biological condition (three replicates) is on the top of the curves. Y-axis represents, for each gene, the number of counts divided by the total number of counts in all the samples (frequency). A: acidic pH and N: neutral pH.

In order to better characterize the different expression profiles of the expressed genes, we performed a co-expression clustering analysis based on Poisson Mixture models [21]. The analysis described 6 different profiles (Fig 3). One profile (cluster 1) showed genes with strong expression in PG6, especially at acidic pH (1,167 genes) and another profile (cluster 2) showed genes with high expression in PG38 at neutral pH (445 genes). Both clusters 1 and 2 were representative of the higher growth rates of both strains according to pH. On the contrary, clusters 3 (1,168 genes) and 4 (503 genes) depicted the genes expressed in PG38 and PG6, respectively, in the pH where their growth were the lowest. In addition, clusters 2, 4, and 6 depicted genes more expressed at neutral pH compared to acidic pH: 445 genes highly expressed in PG38 compared to PG6 (cluster 2), 503 genes, including pacC, highly expressed in PG6 compared to PG38 (cluster 4), and 3,034 genes with similar expression in both strains (cluster 6). As the transcription factor pacC is known to repress acid-expressed genes and to induce neutral-to-alkaline expressed genes [10], the presence of pacC in the cluster 4 confirmed the activation of pH-signaling pathway at neutral pH in our study. The clusters 3 and 5 were more homogeneous between samples whereas cluster 3 showed genes globally more expressed in PG38 and cluster 5 in PG6 (4,724 genes). Interestingly, the clusters 3 and 5 contained the expression of the different pal genes confirming the expression of this pH-signaling pathway in *G*. *tritici* [8].

Finally, the clusters summarized well the different biological growth profiles that took place according to the strains and the pH, as described in the Fig 1, making relevant the study of transcriptional profiles in relation to growth phenotypes.

### Overall comparison of differentially expressed genes (DEGs)

**Fig 4:**
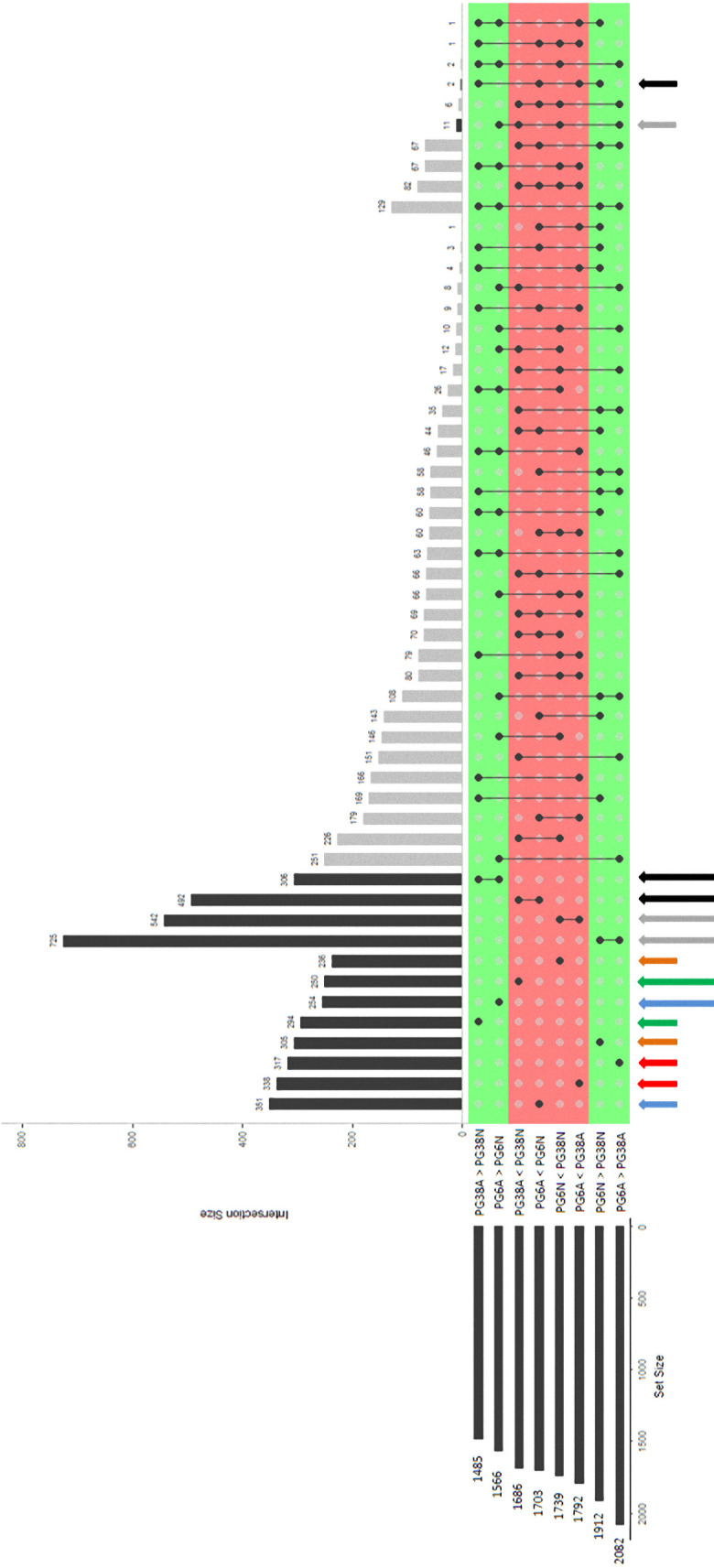
Plot of intersections between sets of genes differentially expressed. The intersected lists of differentially expressed genes and their sizes (number of DEGs) are presented in the horizontal bars on the left. The connected lines among the lists represent their intersections. The vertical bars and associated numbers correspond to specific overlap of DE gene sets. The legends of colored arrows are explained in the text. The significance of gene expression changes was inferred based on an adjusted p-value < 0.05. <: number of DEGs underexpressed in the first condition compared to the second. >: number of DEGs overexpressed in the first condition compared to the second. A: acidic pH and N: neutral pH.

After DESeq2 normalization, four main comparisons biologically relevant were performed: 3,269 genes were differentially expressed between PG6A and PG6N conditions, 3,874 between PG6A and PG38A, 3,171 between PG38A and PG38N, and 3,651 between PG6N and PG38N (**Set Size** on Fig 4). Each of these DEGs lists contained a similar number of genes and represented from 21.5 to 26.3% of the total number of genes of the *G*. *tritici* genome. Among these lists, 605 genes were specific (not found in other comparisons) of the PG6A/PG6N comparison (**blue arrows** on Fig 4), 655 of the PG6A/PG38A contrast (**red arrows** on Fig 4), 544 of the PG38A/PG38N contrast (**green arrows** on Fig 4), and 541 of the PG6N/PG38N contrast (**orange arrows** on Fig 4). Among these four last lists, the percentages of up-regulated and down-regulated were similar and the total number of specific DEGs represented from 3.7 to 4.4% of the total number of genes included in the *G*. *tritici* genome.

The UpSetR package was used to identify 1.267 genes specifically regulated between the two strains independently of the pH (**grey long arrows** on Fig 4 and Tables 3 and S3), and 798 genes regulated between acidic and neutral pH whatever the strain (**black long arrows** on Fig 4 and Tables 3 and S5). The numbers of genes over-expressed in each strain under the pH condition for which each grew best (Table 3) were 486 in PG38 (S7 Table) and 571 in PG6 (S9 Table).

**Table 3:** Number of DEGs according to strain (A), pH (B), and higher growth condition (C).

**A**	Up / Down	Number of DEGs
PG6 compared to PG38 (1,267 genes; S3 Table)	PG38 > PG6	542
	PG6 > PG38	725
**B**	Up / Down	Number of DEGs
Acidic pH compared to neutral (798 genes; S5 Table)	N > A	492
	A > N	306
**C**	Up / Down	Number of DEGs
PG38N (486 genes; S7 Table)	PG38N > PG38A	250
	PG38N > PG6N	236
PG6A (571 genes; S9 Table)	PG6A > PG6N	254
	PG6A > PG38A	317

>: number of DEGs overexpressed in the first condition compared to the second.

A: acidic pH and N: neutral pH.

As both studied factors (strain and pH) had an effect on *G*. *tritici* transcriptome, the study focused on transcriptomics differences between strains whatever the pH (strain effect) on one hand, and on transcriptomics differences between pH whatever the strain (strain effect) on the other hand. Finally, to highlight mechanisms potentially involved in the specific ability of each strain to grow under favorite pH, the overexpressed genes for each strain in the pH condition they grew better were more specifically analysed.

### Strain effect on the *G*. *tritici* transcriptome whatever the pH

**Fig 5:**
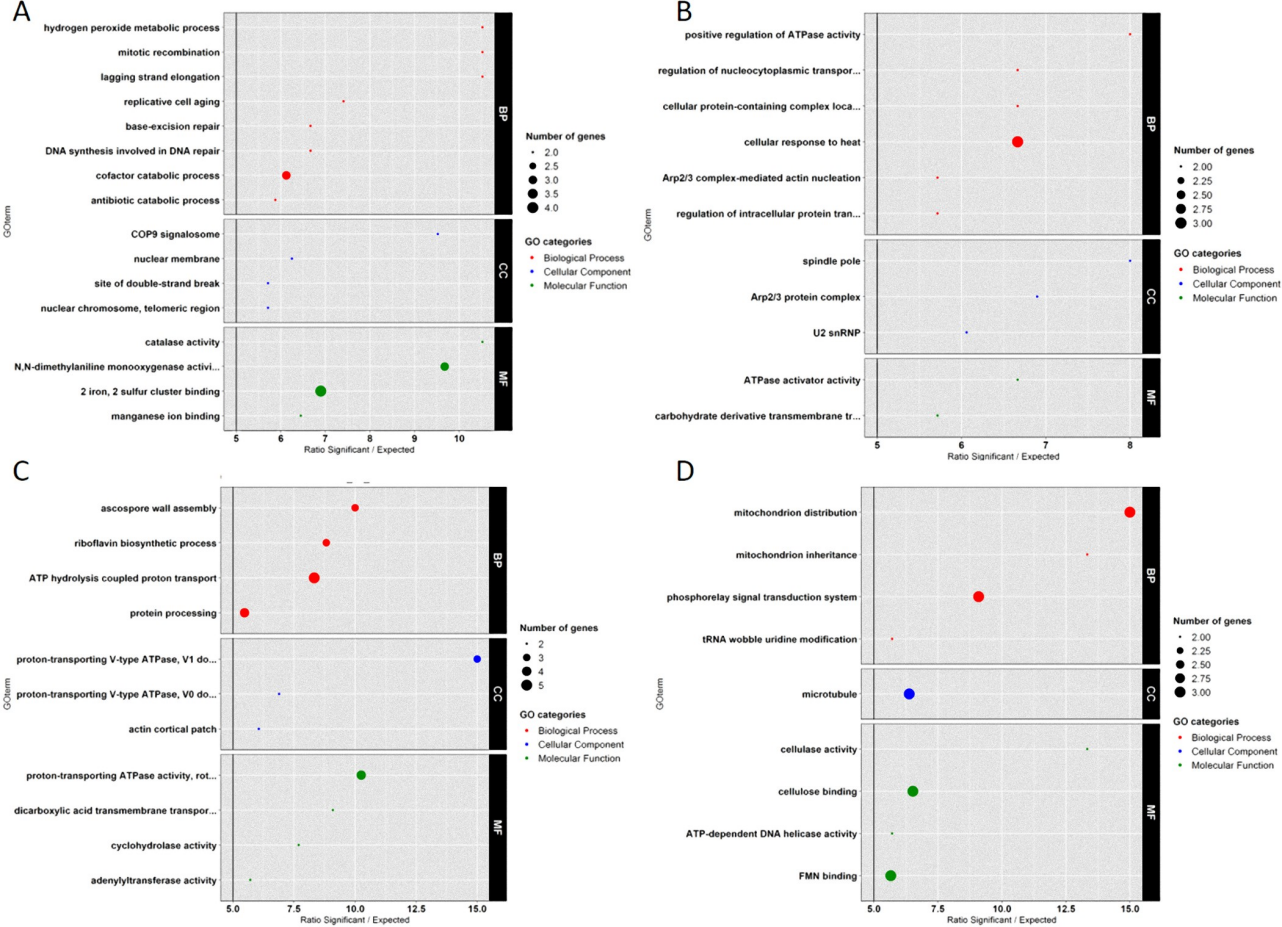
GO enrichment on DEGs lists according to strain or pH effects. Enrichment factor (> 5) is represented on the x-axis and enriched GO-terms are described on the y-axis. (A) Genes linked to strain effect: underexpressed in PG6 compared to PG38. (B) Genes linked to strain effect: overexpressed in PG6 compared to PG38. (C) Genes linked to pH effect: underexpressed in acidic medium compared to neutral. (D) Genes linked to pH effect: overexpressed in acidic medium compared to neutral. BP = Biological Process, CC = Cellular Component and MF = Molecular Function.

To better understand how the transcriptome differed between the strains whatever the pH, a GO-term enrichment analysis was achieved with TopGO. Among the 1,267 DEGs linked to the strain effect (**grey long arrows** on Fig 4 and Table 3), 542 (S3A Table) and 725 (S3B Table) were overexpressed in PG38 and PG6, respectively (Table 3). The Fig 5A and 5B showed that 51 genes were involved in an enrichment of 27 significant GO-terms. Among these 27 enriched GO-terms related to the strain effect, 16 GO-terms were enriched in PG38 (Fig 5A) and 11 in PG6 (Fig 5B).

The genes responsible for the enrichment of the main GO-terms in PG38 are described in S4A Table and encoded proteins involved in DNA synthesis. The GO-terms enriched in PG38 (Fig 5A) were involved in DNA repair and DNA replication mechanisms (‘lagging strand elongation’, ‘replicative cell aging’, ‘base excision repair’, ‘DNA synthesis involved in DNA repair’, and ‘mitotic recombination’). The ‘base excision repair mechanism’ has been especially shown to be involved in host defense in the human pathogenic fungus *Paracoccidioides brasiliensis* [25]. The ‘2 iron, 2 sulfur cluster binding’ term was essential to cell viability in *Saccharomyces Cerevisiae* via its implication in ribosome assembly, DNA damage repair, and DNA replication [26]. In addition, the ‘antibiotic catabolic process’ GO-term was enriched in PG38. In particular, the gene coding for the catalase-1 (S4A Table) was overexpressed. The catalase-1 occurs in almost all aerobically respiring organisms and serves to protect cells from the toxic effects of hydrogen peroxide, a widely used antimicrobial chemical. This mechanism could take part of a global stress-signaling pathway and could, for example, explain a potential ability of this strain to resist to antibiotics synthetized by antagonistic microorganisms in soils [6]. So, whatever the pH, the PG38 seemed to apply DNA repair and stress resistance mechanisms which could be essential in survival during wheat intercrop and growth of *G*. *tritici* in soils and on roots surface.

On the other side, the enriched GO-terms in PG6 (Fig 5B) were associated to mitochondrial inheritance, energy transfer, and energy production. For example, ‘Arp2/3 complex’ was essential to mitochondrial material transport to daughter cells during mitosis [27,28] participating in nucleation of actin filaments. As this transport was also supported by microtubules, the enrichment of ‘spindle pole’ GO-term in PG6 could be important for mitochondrial transport by its action on microtubule organization. The gene encoding ‘nuclear distribution protein pac-1a’ (S4B Table) plays a central role in cell division and cycle. Finally, ‘ATPase activity’ enriched in PG6, suggests an important role for energy transfer. In PG6, a large part of its metabolism seemed to be related to energy mechanisms and cell division potentially important for its growth whatever the pH.

### pH effect on the *G*. *tritici* transcriptome whatever the strain

In the same way, TopGO analysis enabled to identify GO-term enrichments in the genes overexpressed at a given pH compared to the other pH, whatever the strain. Among the 798 genes previously described as linked to the pH effect (**black long arrows** on Fig 4 and Table 3), 492 (S5A Table) and 306 (S5B Table) were respectively overexpressed in neutral and acidic environment. Fig 5C and 5D showed that 53 genes of these DEGs were involved in the enrichment of 20 significant GO-terms. Among these 20 enriched GO-terms linked to the pH effect, 11 GO-terms were enriched in neutral conditions (Fig 5C) whatever the strain and 9 in acidic conditions (Fig 5D).

Several enriched GO-terms in neutral conditions were linked to ‘V-ATPases’ and ‘proton transport’. These pathways have been demonstrated as involved in organelles acidification in cells in response to extracellular pH, with an overexpression at pH 7.0 in yeast cells [29] as for *G*. *tritici* in this study. Under these enriched GO-terms, we found 5 genes (GGTG_01849, GGTG_04071, GGTG_06975, GGTG_04610, and GGTG_10757; S6A Table) expressed at the same level between conditions N and A in both strains and displaying similar fold change (about 1.5) between neutral and acidic conditions. So, both strains of our study seemed to be able, at the same level, to maintain pH-gradients in their cells and organelles when they grew on neutral medium. Enrichment of ‘ascospore wall assembly’ at neutral pH was also seen, suggesting a potential ability of both strains at this pH to form ascospores. As this ability is related to the sexual stage of *G*. *tritici*, which is usually used to disperse at long distance or to resist to stresses, it could have an importance in dynamics of the take-all disease during wheat monocultures, in function of the ambient pH. The genes overexpressed in neutral condition compared to acidic driving the ‘ascospore wall assembly’ GO-term enrichment were 'hypothetical proteins' (S6A Table). However, a blast analysis of the sequences allowed us to refine their potential functions and showed their role in spore wall maturation. They encoded sporulation-specific enzymes involved in the production of a dityrosine-containing precursor of the spore wall (GGTG_03473 and GGTG_03478), and a dityrosine transporter (GGTG_03474) in *Saccharomyces cerevisiae*. No other known genes related to sexual reproduction were enriched in neutral conditions. The analysis of predicted protein—protein interaction with these three genes, performed with the STRING database (https://string-db.org/network/4932.YDR402C), showed that they interacted with the RIM101, which is the yeast equivalent of pacC. The 5’-GCCA(A/G)G-3’ PacC binding consensus was found upstream all the three genes. So, in *G*. *tritici*, an overexpression of these genes involved in spore maturation could be regulated by the pacC transcription factor. This is concordant with the pacC expression pattern found in our study: both strains displayed a similar expression pattern, with pacC transcript levels being highest under neutral growth conditions and lowest under acidic growth conditions. A similar pattern of transcriptional regulation by ambient pH in synthetic medium has been reported in the *pacC* gene from other fungi and also in our previous study [8].

On the other side, the main enriched function at acidic pH was linked to the ‘carbohydrate metabolic process’ (13 genes), probably as a source of nutrients for growth, and to ‘mitochondria transport and inheritance’ (GO-terms ‘mitochondrion distribution’, ‘mitochondrion inheritance’, and ‘microtubule’). As the mitochondria activity could affect various mechanisms such ATP production, cellular differentiation, or cell death [27,28], the role of these enriched genes needs to be elucidated. The gene coding for a threonyl-tRNA synthetase was overexpressed in acidic condition (S6B Table). This enzyme family, involved in the protein translation machinery, is often used as a target in a wide range of antibiotics [30], and its inactivation led to a reduced growth and attenuated virulence in *Leishmania donovani* [31]. In the same way, the regulation of its expression in *G*. *tritici* can be involved in differential growth according to the pH. Finally, the present study confirmed previous data on the expression of some genes involved in pH signal transduction pathway [8]: in PG38 the palB, palC, and palF genes were overexpressed at acidic pH (data nor shown). The mRNA levels of the components of the Pal pathway were then pH-regulated, even if they are usually described as not being [32], showing the expression pattern specificity related to the studied strains.

### Strain and pH effects on *G*. *tritici* transcriptomes according to the growth profiles

To understand more precisely the mechanisms potentially involved in the specific ability of each strain to grow on its favorite pH, we focused on genes overexpressed in each strain in the pH condition they grew better. We previously showed that the *G*. *tritici* strains are able to alkalinize an unbuffered acidic medium with differences between strains in the intensity, the distance, and the persistence of this alcalinization [11]. In the present study, as we checked that the media used herein were unvaryingly buffered at the initial pH over the 7-day experiment, the real effect of the pH can be evaluated on the *G*. *tritici* transcriptomes.

Among the 486 genes specifically overexpressed in PG38 at neutral pH (Fig 4 and Table 3), 250 (S7A Table) and 236 (S7B Table) were overexpressed compared to PG38 in acidic condition and to PG6 in neutral condition, respectively.

**Fig 6:**
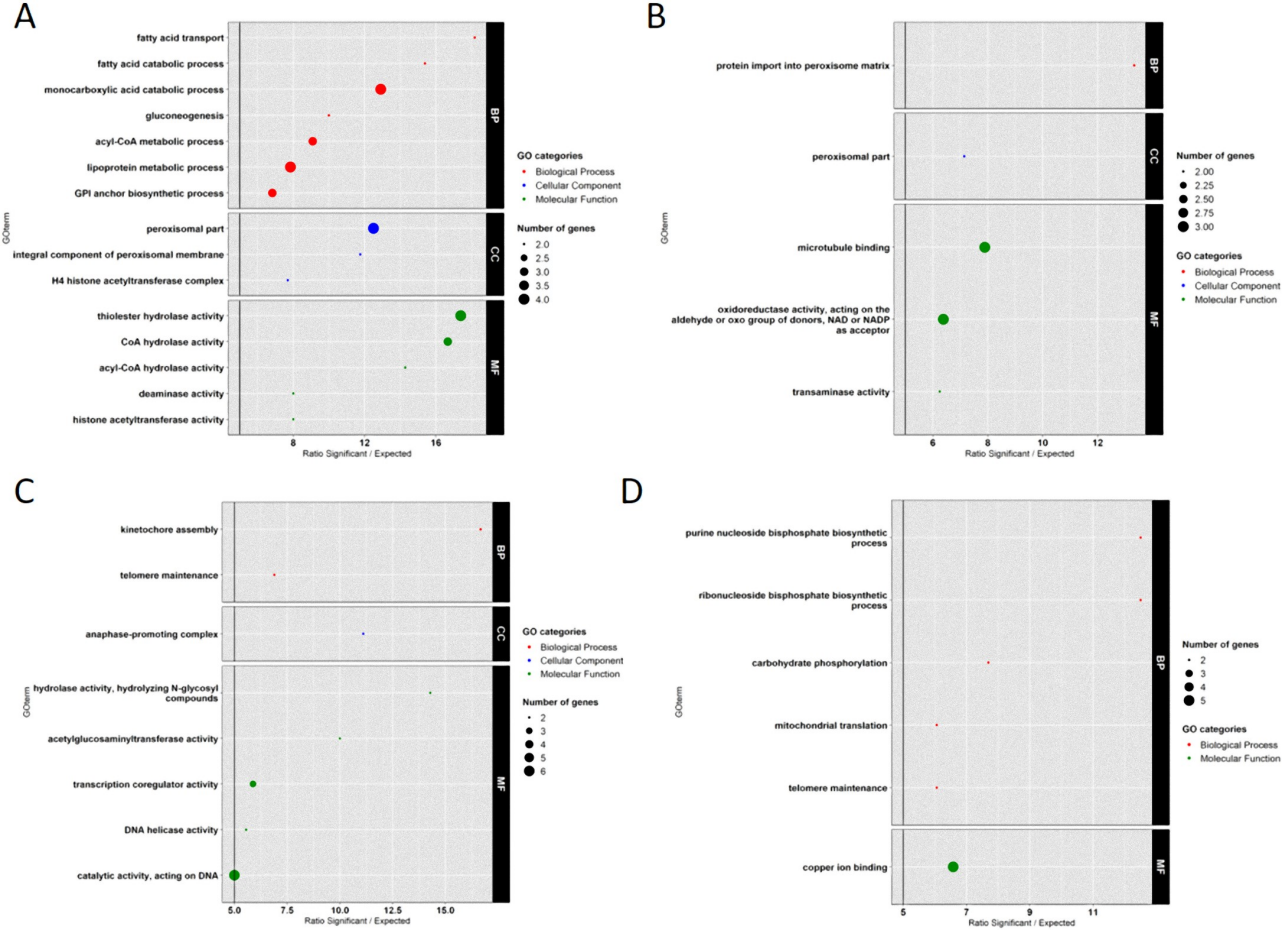
GO enrichment on DEGs explaining growth profiles. Enrichment factor (> 5) is represented on the x-axis and enriched GO-terms are described on the y-axis. (A) 250 DEGs explaining better growth at neutral pH than at acidic pH for PG38. (B) 236 DEGs explaining better growth for PG38 than PG6 at neutral pH. (C) 254 DEGs explaining better growth at acidic pH than at neutral pH for PG6. (D) 317 DEGs explaining better growth for PG6 than PG38 at acidic pH. BP = Biological Process, CC = Cellular Component and MF = Molecular Function.

Thanks to a TopGO identification of enriched GO-terms among the 250 genes overexpressed in PG38 at neutral pH compared to acidic (Fig 6A and **green long arrow** on Fig 4), 41 contributed to enrichment in 15 GO-terms. A main part of the enriched GO-terms (9) was involved in fatty acid metabolism: ‘fatty acid transport’, ‘fatty acid catabolic process’, ‘monocarboxylic acid catabolic process’, ‘acyl-CoA metabolic process’, ‘peroxisomal part’, ‘integral component of peroxisomal membrane’, ‘thiolester hydrolase activity’, ‘CoA hydrolase activity’, and ‘acyl-CoA hydrolase activity’ (S8A Table). As fatty acids are the main source of carbon and energy in fungi suggesting a role in fungal development [33], and as fatty acids uptake and biosynthesis correlated with fungal completion of life cycle [34], this pathway could play a role in the ability of PG38 to better grow at neutral pH. In addition, this strategy seemed to be specific to the growth of PG38 because fatty acid pathways were not enriched in the acidic condition in which PG6 showed better growth (Fig 6C). It has also been shown that lipid metabolism constituted crucial parameters that regulate appressorium formation in the rice blast fungus, suggesting a role in pathogenicity that could be higher in PG38 at neutral pH than at acidic [35].

To better understand why PG38 grew better than PG6 at neutral pH, the 236 genes differentially expressed between both strains at this pH (S7B Table) were also analysed for enriched GO-terms (Fig 6B; 12 contributed to 5 GO-terms enrichment). This analysis highlighted ‘transaminase activity’, ‘oxidoreductase activity’ and ‘peroxisomal part’, for which details of the genes driving enrichment are presented in the S8B Table. Thus, the overexpression of the gene encoding an aromatic amino acid aminotransferase could play a role in the adaptation and survival of PG38 in the changing environment by allowing the growth using different nitrogen sources [36]. In order to survive in different and rapidly changing environments, the fungi must indeed be able to adapt via their expression of genes for amino acid metabolism since amino acids constitute key sources of nitrogen for growth of many fungi. An aminotransferase was also overexpressed in *G*. *tritici* grown on PDA medium [37]. Concerning the genes driving the ‘oxydoreductase activity’ enrichment (S8B Table), study in *Magnaporthe oryzae* showed that the methylmalonate-semialdehyde dehydrogenase regulated pathogenesis [38]. The overexpression of the gene encoding an aldehyde dehydrogenase could play a role in stress response and detoxification [39]. In the same way, the enrichment of the GO-term ‘peroxisomal part’ was explained on one side by a gene encoding the lon protease like 2 protein identified in *M*. *oryzae* to be important for stress resistance and pathogenesis [40], and on the other side by a gene encoding a peroxin 14/17 that was also described as required for full virulence of *M*. *oryzae* [41].

All these functions enriched in PG38 compared to PG6 at neutral pH conditions suggested that the expression of these genes may be crucial in the adaptation PG38 (a G2 strain) to the ambient pH, and were concordant with the higher aggressiveness of G2 strains in *in vitro* plant assays [4]. Specific study of the regulation of these pathogenesis-related genes by the pH-signal transduction pathway would be now interesting.

Concerning PG6 in acidic condition, among the 571 genes specifically overexpressed (Fig 4 and Table 3), 254 (S9A Table) and 317 (S9B Table) were overexpressed compared to PG6 in neutral condition and to PG38 in acidic condition, respectively.

GO-enrichment on the 254 genes overexpressed in PG6 at acidic pH compared to neutral (Fig 6C**)** showed that 21 participated to enrich 8 GO-terms. Three were related to the cell cycle: ‘kinetochore assembly’, ‘telomere maintenance’, and ‘anaphase promoting complex’ **(**S10A Table). Kinetochores are large proteins assemblies that connect chromosomes to microtubules of the mitotic and meiotic spindles [42], and ‘telomere maintenance’ is important for genome stability. The ubiquitin ligase (E3) activity of the ‘anaphase promoting complex’ plays a crucial role division of cells into two daughter cells during mitosis or meiosis [43]. The enrichment in ‘transcription coregulator activity’ showed a high activity of transcription of genes. All these elements might explain in part the higher growth of PG6 at acidic pH, especially through high cell proliferation. Unfortunately, the insufficient annotation of the *G*. *tritici* genome did not allow to go further in interpretation since the majority of the genes explaining these enrichments encoded for ‘hypothetical’ proteins (S10A Table).

In order to study the specific mechanisms in how PG6 was coping with the acidic condition differentially than PG38, GO-enrichment from the 317 genes overexpressed at acidic pH in PG6 compared to PG38 showed that 15 genes participated to enrich 6 GO-terms (Fig 6D). Several GO-terms were linked to general cell proliferation (‘purine nucleoside biphosphate biosynthetic process’, ‘ribonucleoside biphosphate biosynthetic process’) and energy supply (‘mitochondrial translation’, ‘carbohydrate phosphorylation’) which may explain in part the higher growth of PG6 compared to PG38. Moreover, the enrichment of the GO-term ‘copper ion binding’ was driven by 5 genes described in the S10B Table. In *G*. *tritici*, the regulation of genes by copper has been shown, such as laccases. Laccases are multi-copper containing enzymes that catalyze the biological oxidation of various phenolic compounds and *G*. *tritici* laccases may be involved pathogenesis [44]. Driving this GO-term, the genes encoding ‘grisea protein’ and ‘clap1’ were found. Grisea is a copper-modulated transcription factor involved for instance in senescence and morphogenesis from *Podospora anserina* [45]. The clap1 gene was described as encoding a copper-transporting ATPase involved in the process of infection by the phytopathogenic fungus *Colletotrichum Lindemuthianum* [46]. The specific use of copper by strain PG6 under acidic pH conditions compared to strain PG38 is a mechanism that now needs to be further elucidated.

All these analyses gave the first clues as to the mechanisms favored by each strain to better grow at their preferred pH.

### RT-qPCR validation of RNA-Seq analyses

**Fig 7:**
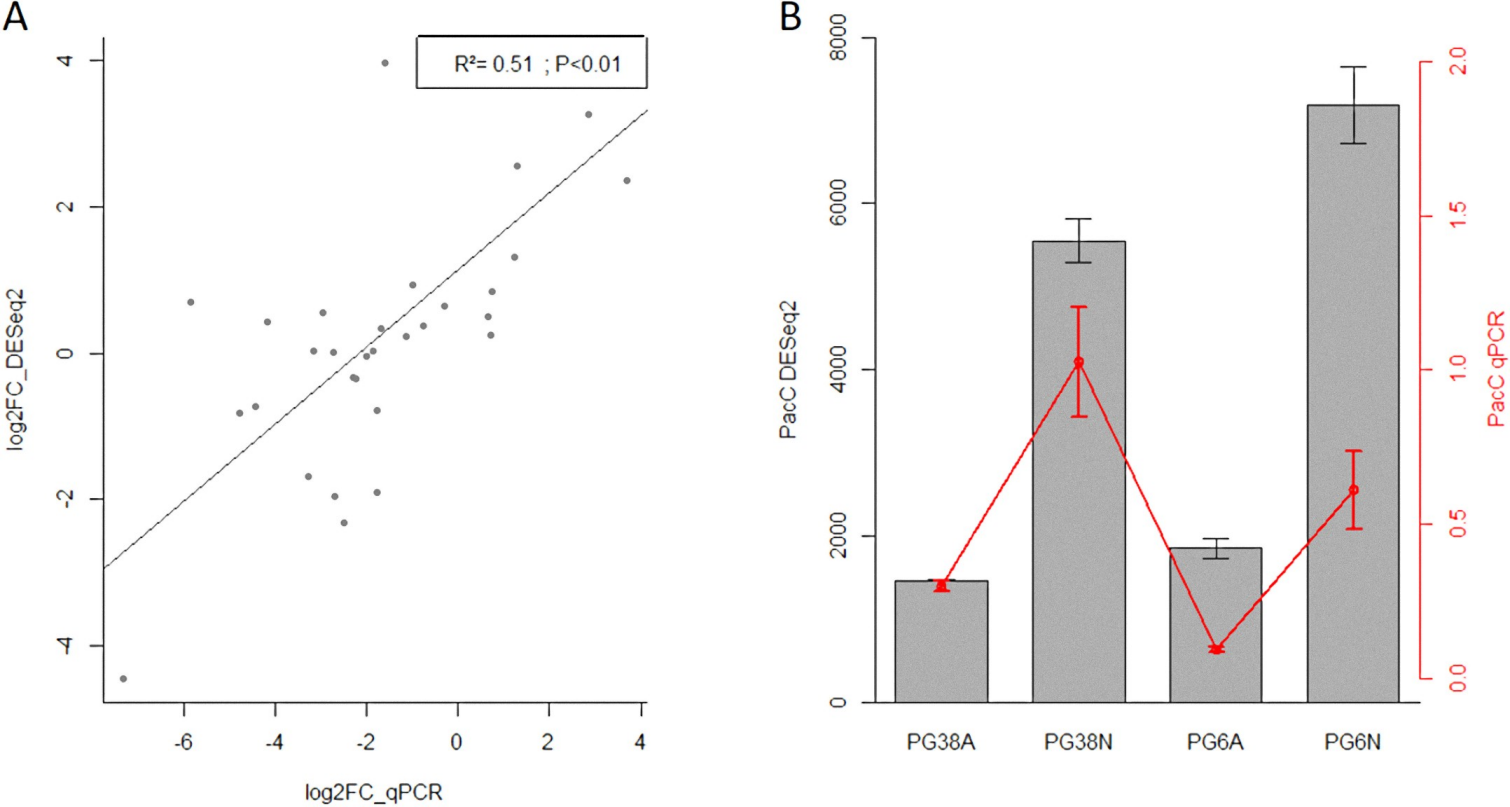
RT-qPCR and RNAseq data comparison. A. Correlation between log2FoldChange of quantitative real-time PCR (x-axis) and RNAseq (y-axis) on 8 selected genes in the four main contrasts. B. Comparison of pacC mean expression in all samples (black bars represent RNAseq results; red points represent RT-qPCR results). Errors bars represent standard errors of the means. A: acidic pH; N: neutral pH; pacC DESeq2: transcript expression of pacC (DESeq2 normalized counts); pacC qPCR: transcript expression of pacC (RT-qPCR expression normalized with external controls).

Eight genes, which were significantly differentially expressed at least twice among the four main comparisons, were selected to validate the RNA-Seq data by RT-qPCR assay. RT-qPCR primers are described in S2 Table. The selected genes take part of the Pal pathway (palB, palC, palF, and pacC) or are related to pathogenesis (lac1, lac2, gmk1, and exo) [8]. This panel provided a large range of expression levels and enabled us to compare log2FoldChange values between RT-qPCR and RNA-Seq experiments. We found a moderate correlation (r^2^ = 0.51) between log2FoldChanges of both types of experiments but with a very strong significance according to Pearson correlation test (p-value < 0.01), confirming the validity of the RNA-Seq data (Fig 7A). The similarity of the data between RT-qPCR and RNA-Seq was more precisely confirmed by comparing the pacC (GGTG_01809) expression (Fig 7B). In both types of experiments, pacC displayed similar expression’s pattern: it was less expressed at acidic pH than at neutral pH, as observed in other fungi such as *A*. *nidulans* [47], *Sclerotinia sclerotiorum* [48], *Fusarium Oxysporum* [49], *Colletotrichum acutatum* [50], *Aspergillus oryzae* [51], or *Coniothyrium minitans* [52].

Although slight variations in correlations were observed between the two methods, similar trends in transcript abundances were generally observed confirming a reliable expression result generated by RNA sequencing.

## Conclusions

This study provides new insights on gene expression regulation monitored by the ambient pH in a strain-specific way with opposite growth rate profiles in function of the extracellular pH.

Independently of the ambient pH, the two strains have different strategies in adapting transcriptome to growth: PG38 expressed mainly genes involved in response to stresses (DNA repair and antibiotic resistance), whereas genes involved in mitochondrial inheritance and energy transfer were more expressed in PG6. So PG38 (G2 type) could have a better ability to survive and/or to resist to stresses, whereas PG6 (G1 type) could have a better ability to grow. Moreover, both strains are able to regulate their intracellular pH and activated mechanisms to produce ascospores when growing in neutral conditions whereas they overexpressed functions linked to mitochondria transport in acidic conditions.

This study also highlighted that both strains adopted different strategies to grow, depending on the ambient pH. These two strains were able to grow on both pH conditions. At acidic pH, PG6 was able to highly grow and showed an enrichment of pathways related to cell division and proliferation, but no genes involved in survival strategies were particularly expressed. On the other hand, PG6 was disadvantaged at neutral pH because it had lower ability to grow and to survive or resist to stresses. These two characteristics could finally lead to a decrease of this type of strains in fungal population. Concerning PG38, the strain had better growth in neutral conditions, especially via fatty acids and amino acids utilization. At acidic pH, despite a lower growth, this strain displayed ability to stress resistance potentially linked to survivability. In total, whatever the pH but particularly at neutral pH, PG38 could have better abilities to survive in soils during intercrops, so allowing the presence of inoculum source to the next wheat culture. Interestingly, PG38 overexpressed at neutral pH genes potentially involved in pathogenicity, even in the absence of a plant. This is consistent with the greater aggressiveness of G2 strains, the group to which PG38 belongs. Thus, we described, in an original way, different pH-dependent strategies related to growth rate profiles in the two studied strains. Additional studies (e.g. gene inactivation) are necessary to explore if some proteins encoded by interesting DEGs have expected functions.

To go further in the understanding of survival and infection dependent on the pH and on the strain, particularly concerning the peak of G2 type after few years of wheat monoculture in neutral conditions, other similar studies including more strains and pH conditions are necessary. Moreover, *G*. *tritici* transcriptomics study in host plant grown in soils of different pH would be interesting to focus more precisely on the infection stage. At this time indeed, only one transcriptomics studies were performed in *G*. *tritici* [37,53], and one highlighted that over 3,000 genes (including genes involved in signal transduction pathways, development, plant cell wall degradation, and response to plant defense compounds) were differentially expressed between *G*. *tritici* in culture and *G*. *tritici* infecting roots, but this study is based on a single strain not characterized for G1/G2 type [53].

## Supporting information

S1 Fig Estimation of biological variations by a Principal Component Analysis of the 12 transcript profiles. X-axis represent the variance on the first axis of the PCA, and the second is picted on the y-axis. A: acidic pH and N: neutral pH
(PDF)

S1 Table Composition of the media used in this study. (PDF)

S2 Table Oligonucleotide primers used in this study. (PDF)

S3 Table Description of the *G*. *tritici* genes differentially expressed between the strains. (XLSX)

S4 Table Description of the gene subsets responsible for the main driving GO-term enrichments between the strains. (XLSX)

S5 Table Description of the *G*. *tritici* genes differentially expressed between the pH. (XLSX)

S6 Table Description of the gene subsets responsible for the main driving GO-term enrichments between the pH. (XLSX)

S7 Table Description of the overexpressed *G*. *tritici* genes in PG38N explaining better growth of PG38 at neutral pH. (XLSX)

S8 Table Description of the gene subsets responsible for the main driving GO-term enrichments explaining better growth of PG38 at neutral pH. (XLSX)

S9 Table Description of the overexpressed *G*. *tritici* genes in PG6A explaining better growth of PG6 at acidic pH. (XLSX)

S10 Table Description of the gene subsets responsible for the main driving GO-term enrichments explaining better growth of PG6 at acidic pH. (XLSX)
