# Supplementary material for: pH effect on strain-specific transcriptomes of the take-all fungus
Source: PLoS One. 2020 Jul 30;15(7):e0236429. doi: 10.1371/journal.pone.0236429 (PMC7392285; doi:10.1371/journal.pone.0236429)
Supplement: S1 Fig — X-axis represent the variance on the first axis of the PCA, and the second is picted on the y-axis. A: acidic pH and N: neutral pH (PDF) [file pone.0236429.s001.pdf]

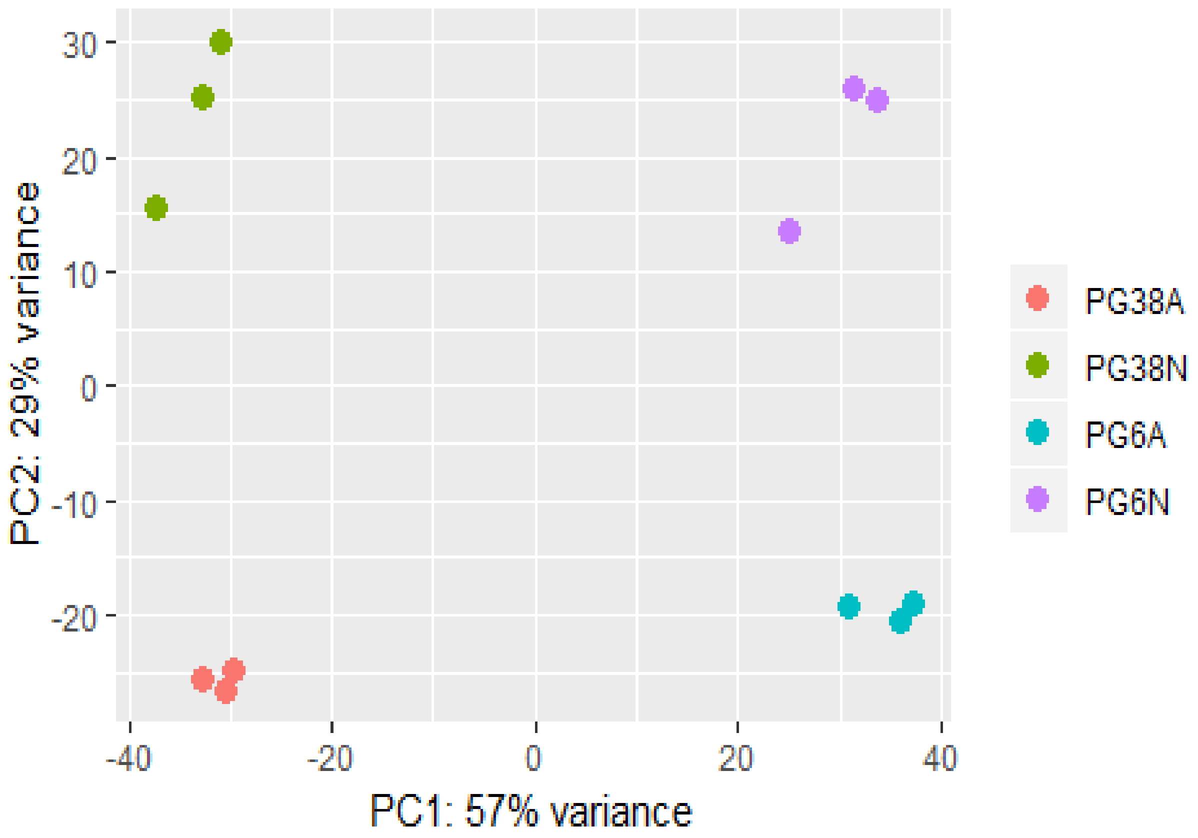

S1 Fig. Estimation of biological variations by a Principal Component Analysis of the 12 transcript profiles.
X-axis represented the variance on the first axis of the PCA, and the second was picted on the y-axis.
A: acidic pH and N: neutral pH
