# Supplementary material for: pH effect on strain-specific transcriptomes of the take-all fungus
Source: PLoS One. 2020 Jul 30;15(7):e0236429. doi: 10.1371/journal.pone.0236429 (PMC7392285; doi:10.1371/journal.pone.0236429)
Supplement: S1 Table — (PDF) [file pone.0236429.s002.pdf]

S1 Table. Composition of the media used in this study.

	Product	Final Concentration	
Fahraeus medium	Product	Final Concentration	
	Glucose	110 mM	
	L-Asparagin	19 mM	
	D,L-Phenylalanin	0.90 mM	
	Adenin	0.20 mM	
	Thiamin-HCl	0.15 µM	
	KH_2PO_4	7.30 mM	
	Na_2HPO4	0.56 mM	
	$MgSO_4$	2 mM	
	$CaCl_2$	0.09 mM	
	$FeSO_4$	0.03 mM	
	$MnSO_4$	4.50 µM	
	$ZnSO_4$	3.50 µM	
	$CuSO_4$	8 µM	
	Biotin	1 µM	
	Agar	1.50 % (w/v)	
Citrate / Phosphate Buffer	Product	Volume for 1 L	Final pH
	Citrate 0.2 M	537 mL	4.6
	Disodique phosphate 0.1 M	463 mL	
	Citrate 0.2 M	190 mL	7.0
	Disodique phosphate 0.1 M	810 mL	
