# Supplementary material for: pH effect on strain-specific transcriptomes of the take-all fungus
Source: PLoS One. 2020 Jul 30;15(7):e0236429. doi: 10.1371/journal.pone.0236429 (PMC7392285; doi:10.1371/journal.pone.0236429)
Supplement: S2 Table — (PDF) [file pone.0236429.s003.pdf]

S2 Table. Oligonucleotide primers used in this study

Targeted gene [a]	Source [b] Broad Institute / GenBank	Forward primer 5’ - 3’	Reverse primer 5’ - 3’	Expected amplicon size (bp)	Annealing temperature (°C)
pacC	GGTG_01809	caagcgcgatcacatcacct	gagaaccgagtcgtcggc	139	57
palB	GGTG_09525	gcagcaggacatctttgcag	gcggcatcaaagggatacat	243	63
palC	GGTG_06037	aagaagagcggcttccgttt	ggtatcattttgcttggaccatt	165	58
palF	GGTG_01551	atgactatggggcaatggggt	ctccagcttaggtcgaagcgaa	150	60
lac1	AJ417685/GGTG_02417	cgctcgagaggatctttgac	gtggatggggtgggtgag	146	62
lac2	AJ417686/GGTG_02239	ttccgctggtacctcaactc	atgacgtaggcccacttgtc	143	62
gmk1	AF258529/GGTG_05241	agaagctcctggccttcaac	ttgctgaggttgtccttgtg	160	60
exo	CF554536/GGTG_01690	gcgggactggagtactacgg	gggtcctgccagatggagat	146	60
Wn1	Sabater-Muñoz et al., 2006	tcatttccttctgggcattc	gcacaaaacactgccatcaa	146	61
Wn2	Sabater-Muñoz et al., 2006	gtaacgtcgtcccactttcaat	ctaacgtcgggcgtaaactaat	140	51

[a] *lac1*, laccase 1; *lac2*, laccase 2; *gmk1*, mitogen-activated protein (MAP) kinase; *exo*, β-1,3-exoglucanase; *Wn1*, 3’ *Wunen* EST region amplified in qRT-PCR; *Wn2*, 5’ *Wunen* EST region amplified in qRT-PCR (Daval et al., 2013).

[b] http://www.broadinstitute.org/annotation/genome/magnaporthe_comparative/GenomesIndex.html, GenBank accession number, or reference [24].
